# Genome-Wide Identification of the YABBY Gene Family in *Dendrobium* Orchids and Its Expression Patterns in *Dendrobium chrysotoxum*

**DOI:** 10.3390/ijms241210165

**Published:** 2023-06-15

**Authors:** Qinyao Zheng, Xuewei Zhao, Ye Huang, Meng-Meng Zhang, Xin He, Shijie Ke, Yuanyuan Li, Cuili Zhang, Sagheer Ahmad, Siren Lan, Minghe Li, Zhong-Jian Liu

**Affiliations:** 1 Key Laboratory of National Forestry and Grassland Administration for Orchid Conservation and Utilization at College of Landscape Architecture, Fujian Agriculture and Forestry University, Fuzhou 350002, China; 2 College of Forestry, Fujian Agriculture and Forestry University, Fuzhou 350002, China

**Keywords:** YABBY transcription factor, *Dendrobium* orchid, expression pattern, *D. chrysotoxum*, *D. huoshanense*, *D. nobile*

## Abstract

The small plant-specific YABBY gene family plays key roles in diverse developmental processes in plants. *Dendrobium chrysotoxum*, *D. huoshanense*, and *D. nobile* are perennial herbaceous plants belonging to Orchidaceae with a high ornamental value. However, the relationships and specific functions of the YABBY genes in the *Dendrobium* species remain unknown. In this study, six *DchYABBY*s, nine *DhuYABBY*s, and nine *DnoYABBY*s were identified from the genome databases of the three *Dendrobium* species, which were unevenly distributed on five, eight, and nine chromosomes, respectively. The 24 YABBY genes were classified into four subfamilies (CRC/DL, INO, YAB2, and FIL/YAB3) based on their phylogenetic analysis. A sequence analysis showed that most of the YABBY proteins contained conserved C2C2 zinc-finger and YABBY domains, while a gene structure analysis revealed that 46% of the total YABBY genes contained seven exons and six introns. All the YABBY genes harbored a large number of Methyl Jasmonate responsive elements, as well as anaerobic induction *cis*-acting elements in the promoter regions. Through a collinearity analysis, one, two, and two segmental duplicated gene pairs were identified in the *D. chrysotoxum*, *D. huoshanense*, and *D. nobile* genomes, respectively. The *Ka/Ks* values of these five gene pairs were lower than 0.5, indicating that the *Dendrobium* YABBY genes underwent negative selection. In addition, an expression analysis revealed that *DchYABBY2* plays a role in ovary and early-stage petal development, while *DchYABBY5* is essential for lip development and *DchYABBY6* is crucial for early sepal formation. *DchYABBY1* primarily regulates sepals during blooming. Furthermore, there is the potential involvement of *DchYABBY2* and *DchYABBY5* in gynostemium development. The results of a comprehensive genome-wide study would provide significant clues for future functional investigations and pattern analyses of YABBY genes in different flower parts during flower development in the *Dendrobium* species.

## 1. Introduction

The small YABBY gene family, belonging to the zinc-finger superfamily, is specific to seed plants. As transcriptional regulators, YABBY genes play pivotal roles in lateral organ development [1], the stress response [2], phytohormone synthesis [3], adaxial–abaxial polarity establishment, [4] and leaf margin establishment [5]. The members of this family possess two highly conserved DNA-binding domains: an N-terminal Cys2Cys2 zinc-finger motif and a C-terminal helix–loop–helix YABBY domain [6,7]. These two structural domains have been shown to be implicated in the specific binding of DNA [6]. Evolutionary analysis has indicated that the YABBY genes in angiosperms can be divided into five groups, including INNER NO OUTER (INO), CRABS CLAW (CRC), YABBY2 (YAB2), FILAMENTOUS FLOWER (FIL)/YABBY3 (YAB3), and YABBY5 (YAB5) [8,9,10,11].

YABBY genes have been well identified and analyzed in the model plant *Arabidopsis thaliana*. *AtFIL*/*AtYAB3*, *AtYAB2*, and *AtYAB5* are highly expressed in vegetative tissues, whereas *AtCRC* and *AtINO* are restricted to the reproductive organs, and are thus called floral-specific YABBY genes [12,13,14]. *AtFIL* is necessary for floral meristem identity establishment and flower development [15]. *AtCRC* is involved in the development of nectaries [8] and the apical end of the carpel [11,16], while *AtINO* expresses in the outer integument of ovules [17]. Isolated on the basis of homology to *AtCRC*, three YABBY genes (*AtFIL*, *AtYAB2,* and *AtYAB3*) play important roles in the specification of abaxial cell fate in lateral organs [18]. Furthermore, YABBY genes have been found to be involved in the developmental processes of many core eudicots. *SlCRCa*, a CRC homolog from the tomato (*Solanum lycopersicum*), has high expression in the petals and stamens [19], while *VvYAB1*, *2*, *3,* and *5* have high expression levels in the vegetative organs of grapes (*Vitis vinifera*) [20]. As for monocots, members of the YABBY family have been well reported in rice (*Oryza sativa*). Molecular cloning has revealed that *OsDL* (DROOPING LEAF), closely related to *AtCRC*, is a member of the YABBY gene family, which regulates midrib formation by promoting cell proliferation in the central region of the rice leaf [21]. *OsYABBY1* is involved in regulating the differentiation of a few specific cell types, rather than the polar regulation of lateral organ development [10]. *OsYABBY4*, belonging to the FIL/YAB3 subfamily, expresses in the meristems and developing vascular tissue of rice, predominantly in the phloem tissues [9].

Orchidaceae is one of the largest and most widespread families in the flowering plant kingdom, with more than 28,000 species [22]. As the second-largest genus of Orchidaceae, *Dendrobium* has been extensively studied for its medicinal properties and ornamental value, and has deeply fascinated botanists and plant enthusiasts over the centuries [23]. Although YABBY genes have been widely reported in model plants and main crops, nothing is known about the relationship and specific functions of the YABBY genes in the *Dendrobium* species during flower development. In recent years, some species of *Dendrobium,* including *D. chrysotoxum* [24], *D. huoshanense* [25], and *D. nobile* [26], have been sequenced. A high-quality, chromosomal-level assembly of the whole genome of these *Dendrobium* species would provide a valuable database resource, facilitating the systematic exploration of the YABBY gene family in orchids.

In this study, 24 *Dendrobium* YABBY genes were identified using a bioinformatics analysis method, including gene structure, motif composition, chromosomal localization, phylogenetic tree analysis, and segmental duplication analysis. The expression patterns of *D. chrysotoxum* were analyzed. The results provide useful information on the biological function of YABBY genes in *Dendrobium* and the molecular mechanisms underlying floral morphogenesis in *D. chrysotoxum*.

## 2. Results

### 2.1. Screening of DchYABBY, DhuYABBY, and DnoYABBY Transcription Factors

Using six *Arabidopsis* YABBY protein sequences as the queries, 26 YABBY genes were initially identified by BLAST searches. These candidate genes were uploaded to Simple HMM search, NCBI, and SMART for further confirmation. Finally, six *DchYABBY*s, nine *DhuYABBY*s, and nine *DnoYABBY*s were obtained as YABBY family members. According to the order distribution on the chromosomes, a total of 24 YABBY genes were named *DchYABBY1*–*6*, *DhuYABBY1*–*9*, and *DnoYABBY1*–*9*. In addition, an encoded protein sequence analysis indicated that the physicochemical properties, including aa, pI, MW, GRAVY, II, and AI, of the YABBY genes in *Dendrobium* were considerably different. The shortest was *DhuYABBY6* with 95 aa and the longest were *DhuYABBY5* and *DnoYABBY2* with 233 aa. The pI values of the 24 YABBY genes in *Dendrobium* ranged from 5.74 (*DnoYABBY3*) to 10.00 (*DhuYABBY6*). Among them, eight YABBY proteins had an acidic pI below seven, while sixteen YABBY proteins with a pI higher than seven were alkaline. The MW values ranged from 10,677.48 (*DchYABBY4*) to 26,103.1 kDa (*DnoYABBY2*), with an average MW value of 20,446.78 kDa. Most of the YABBY proteins showed a GRAVY value less than 0, suggesting that they were hydrophilic. The II values ranged from 27.48 (*DchYABBY2*) to 61.40 (*DhuYABBY2*), with the AI values between 49.20 (*DhuYABBY9*) and 97.42 (*DchYABBY4*). The physicochemical properties for the related YABBY genes are shown in Supplementary Table S1.

### 2.2. Phylogeny and Classification of YABBY Genes

**Figure 1:**
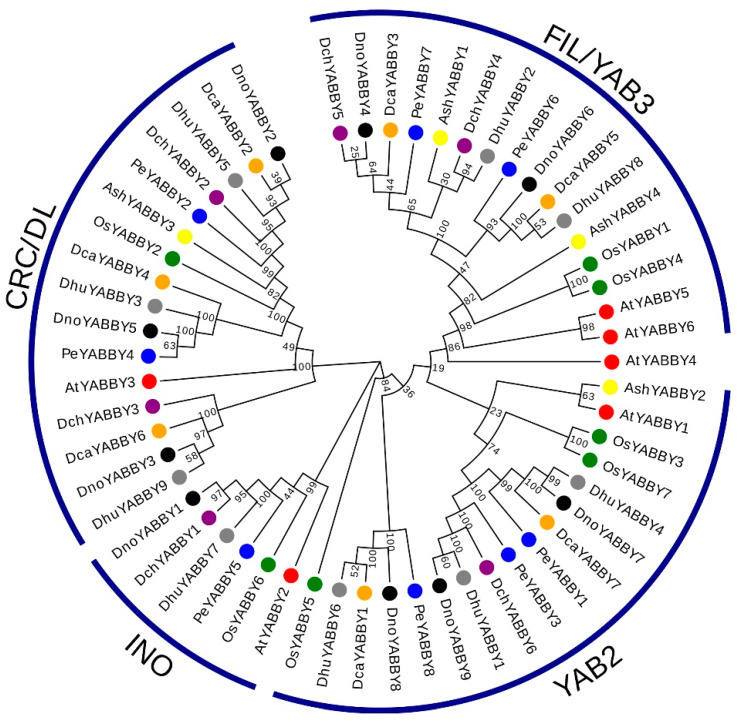
Phylogenetic tree of the 56 YABBY proteins from *D. chrysotoxum*, *D. huoshanense*, *D. nobile*, *A. thaliana*, *O. sativa* subsp. *Indica*, *A. shenzhenica*, *D. catenatum*, and *P. equestris*. The phylogenetic tree was constructed with the neighbor-joining (NJ) method in MEGA 7.0 software and was divided into four subfamilies according to the classification of *AtYABBY*s.

To analyze the deeper relationships and roles of YABBY family members in *Dendrobium*, we used 56 YABBY protein sequences from *D. chrysotoxum*, *D. huoshanense*, *D. nobile*, *A. thaliana*, *O. sativa* subsp. *Indica*, *A. shenzhenica*, *D. catenatum*, and *P*. *equestris* to construct a neighbor-joining (NJ) phylogenetic tree (Figure 1). Based on the classification of *AtYABBY*s, the 24 YABBY genes in *Dendrobium* were divided into four subfamilies, named CRC/DL, INO, YAB2, and FIL/YAB3. The CRC/DL subfamily had the largest number of members (a total of eight members, three *DnoYABBY*s, three *DhuYABBY*s, and two *DchYABBY*s), while the INO subfamily harbored the least members (three members, *DnoYABBY1*, *DhuYABBY7*, and *DchYABBY1*). Seven *Dendrobium* YABBY proteins were exhibited in the YAB2 subfamily, followed by the FIL/YAB3 subfamily (six members).

### 2.3. Gene Structure and Motif Analysis of YABBY Genes

**Figure 2:**
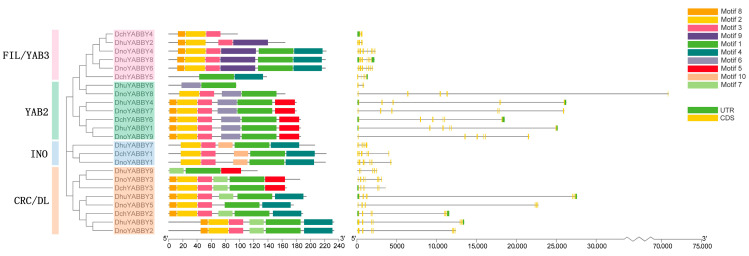
The overall comparative YABBY genes map of the NJ phylogenetic tree, conserved protein motifs, and gene structure.

To observe the gene structure of the YABBY genes in *Dendrobium*, ten conserved motifs were predicted through the MEME program, and the exon–intron structures were exhibited using Tbtools (Figure 2). The results show that most of the YABBY genes had four conserved motifs in the order of motifs 8, 2, 3 and 1. In addition, YABBY family members in the same subfamily had similar conserved motifs, and had certain specific motifs which were not found in the other subfamilies. Motif 9 was observed only in FIL/YAB3 and motif 6 was peculiar to YAB2. The INO subfamily possessed motif 10 exclusively and motif 7 only existed in the CRC/DL clade. Interestingly, motif 5 was present in two clades (YAB2 and CRC/DL), while motif 4 appeared in all clades except the YAB2 clade. Further, almost all the YABBY genes harbored at least four conserved motifs, while *DchYABBY4* and *DhuYABBY9* contained three conserved motifs, and *DchYABBY5* and *DhuYABBY6* had only two motifs.

Subsequently, all 24 YABBY genes possessed introns ranging from one to six. Among them, *DnoYABBY8* had the longest intron, followed by *DhuYABBY3*. In general, 46% of the total YABBY genes (11 members) contained seven exons and six introns, while 33% (eight genes) had six exons and five introns.

**Figure 3:**
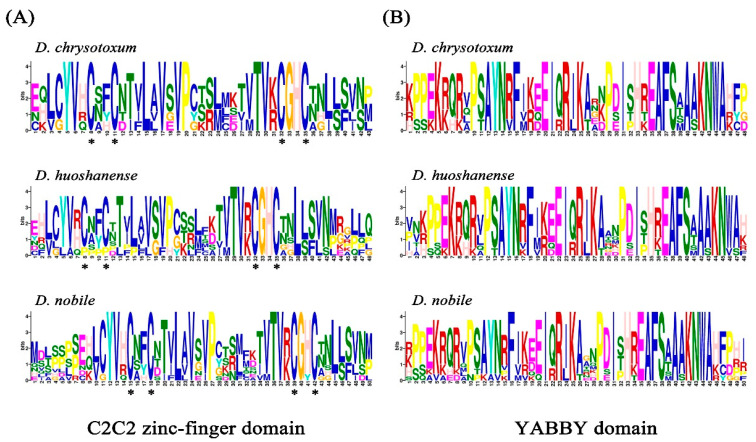
Conserved domains of the three *Dendrobium* species protein sequences. (**A**) Sequence logo of the C2C2 zinc-finger domain. (**B**) Sequence logo of the YABBY domain (* indicates highly conserved cysteine residues).

Sequence logos of the C2C2 zinc-finger and YABBY domains in the three *Dendrobium* species were generated by multiple sequence alignments. As shown in Figure 3, these two domains were highly conserved. The C2C2 zinc-finger domain (Figure 3A) had serval significantly conserved amino acid residues, including cysteine residues (C), glycine residues (G), histidine residues (H), and valine residues (V). In the YABBY domain (Figure 3B), most of the amino acid residues were extremely conserved.

### 2.4. Promoter Analysis of YABBY Genes

**Figure 4:**
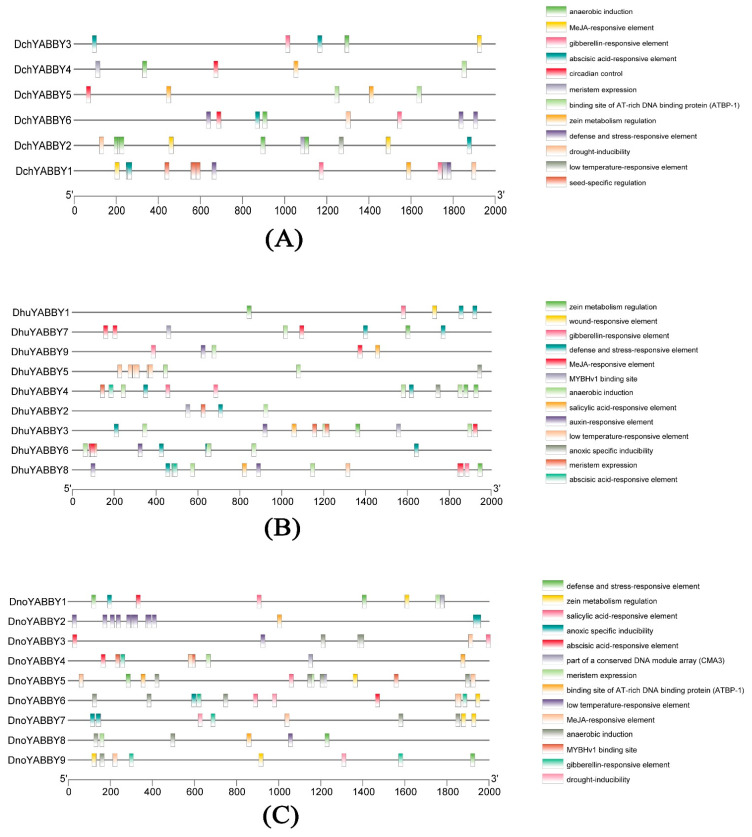
Regulatory elements in the promoter region of the three *Dendrobium* species. (**A**) The *cis*-acting elements of *D. chrysotoxum*. (**B**) The *cis*-acting elements of *D. huoshanense*. (**C**) The *cis*-acting elements of *D. nobile*. The raw data are listed in Supplementary Table S2.

A large number of *cis*-acting regulatory elements in the promoters of *DchYABBY*s, *DhuYABBY*s, and *DnoYABBY*s were obtained. The most frequently occurring responsive elements in the upstream sequence of *D. chrysotoxum* included Methyl Jasmonate (MeJA)-responsive elements (8 times), anaerobic induction (ARE)-responsive elements (8 times), and abscisic acid-responsive elements (7 times) (Figure 4A). However, a low temperature-responsive element only appeared once in *DchYABBY2*. It is noteworthy that all four seed-specific regulation elements appeared in the 400–600 bp upstream CDS of *DchYABBY1*. In Figure 4B, similar to the *cis*-acting regulatory elements in *D. chrysotoxum*, both the MeJA-responsive elements and ARE elements were observed most frequently. *DhuYABBY8* had the largest number of *cis*-acting regulatory elements (14), followed by *DhuYABBY4* (12). *DhuYABBY2* contained the least number with only four elements. A wound-responsive element was solely present in *DhuYABBY1*. The largest number of elements were found in *DnoYABBY5* (fourteen elements), while *DnoYABBY8* had the fewest (six elements) (Figure 4C). Interestingly, low temperature-responsive elements mainly appeared in the 0–500 bp upstream CDS of *DnoYABBY2*. Generally, all three *Dendrobium* species had AREs, MeJA-responsive elements, gibberellin-responsive elements, abscisic acid-responsive elements, meristem expression, zein metabolism regulation, and low temperature-responsive elements, suggesting the wide functional variability of the YABBY genes in *Dendrobium*.

### 2.5. Chromosomal Localization of YABBY Genes

**Figure 5:**
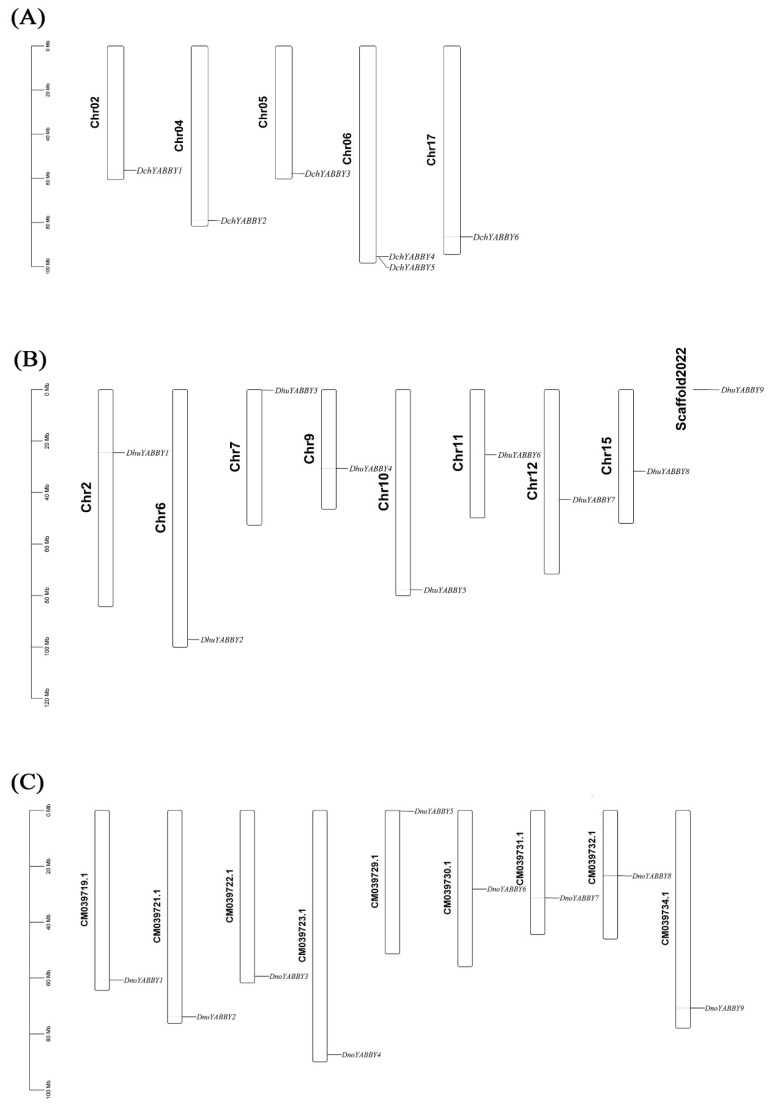
Chromosome distribution in the three *Dendrobium* species. (**A**) Chromosome distribution in *D. chrysotoxum*. (**B**) Chromosome distribution in *D. huoshanense*. (**C**) Chromosome distribution in *D. nobile*.

As shown in Figure 5A, six *DchYABBY*s were distributed on five chromosomes of *D. chrysotoxum* (Chr02, 04, 05, 06, and 17). Chromosome 06 had two genes (*DchYABBY4* and *DchYABBY5*), while the other chromosomes each contained one gene. The chromosome mapping results for *D. huoshanense* indicated that eight *DhuYABBY*s were unevenly distributed on Chromosomes 2, 6, 7, 9, 10, 11, 12, and 15 (Figure 5B). *DhuYABBY9* was localized to the unanchored scaffold, named Scaffold2022. Nine *DnoYABBY*s were present on nine chromosomes. Only *DnoYABBY5* was localized on the top of CM039729.1 (Figure 5C).

### 2.6. Collinearity Analysis and Ka/Ks Value of YABBY Genes

**Figure 6:**
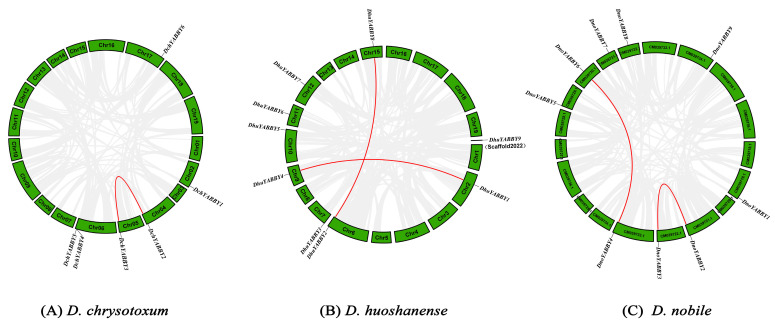
Synteny analysis of the YABBY genes in the three *Dendrobium* species. (**A**) Synteny analysis of *DchYABBY* genes. (**B**) Synteny analysis of *DhuYABBY* genes. (**C**) Synteny analysis of *DnoYABBY* genes. Red lines represent segmental duplicated gene pairs.

The *D. chrysotoxum* genome harbored one pair of segment duplicated genes, which were *DchYABBY2* on Chr04 and *DchYABBY3* on Chr05 (Figure 6A). Two segmental duplicated gene pairs were identified in the *D. huoshanense* genome, which were *DhuYABBY1* on Chr2 and *DhuYABBY4* on Chr9, and *DhuYABBY2f* on Chr6 and *DhuYABBY8* on Chr15 (Figure 6B). Similarly, the *D. nobile* genome also contained two pairs of segmental duplicated genes (Figure 6C), *DnoYABBY2* and *DnoYABBY3*, and *DnoYABBY4* and *DnoYABBY6,* which showed similar conserved motifs and gene arrangements.

The *Ka/Ks* values of all five gene pairs were lower than 0.5, ranging between 0.1 and 0.46. The average *Ka/Ks* value of *D. huoshanense* was 0.24, lower than that of *D. nobile* (0.27) (Supplement Table S3).

### 2.7. Expression Patterns of YABBY Genes in D. chrysotoxum

**Figure 7:**
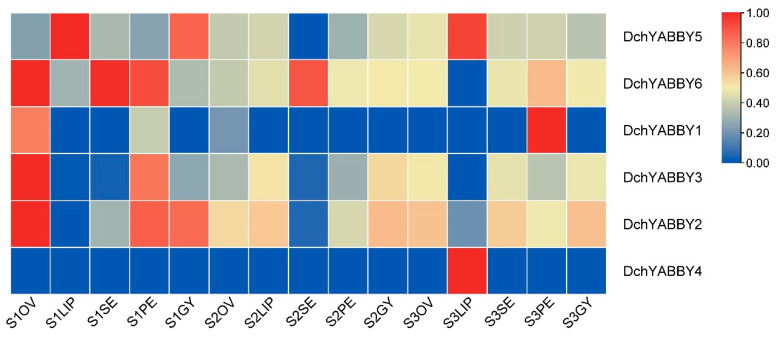
The expression levels of six *DchYABBY*s in different parts and developmental periods in *D. chrysotoxum*. S1: unpigmented bud stage; S2: pigmented bud stage; S3: fully opened flower stage; Ov: ovary; LIP: lip; SE: sepal; PE: petal; GY: gynostemium. The FPKM values of the YABBY genes in *D. chrysotoxum* are listed in Supplementary Table S4.

According to the FPKM values, the expression levels of six *DchYABBY*s in different parts and developmental periods (Figure 7) suggest the higher expression of *DchYABBY2*, *3*, *5*, and 6 in the S1 stage than in the other two stages. *DchYABBY1* showed high expression in the ovary in the S2 stage, and *DchYABBY4* was only expressed in the lip in the S3 stage. *DchYABBY2* and *DchYABBY3*, both belonging to the CRC/DL subfamily, exhibited similar expression patterns throughout flower development. *DchYABBY5* was highly expressed in the lip and gynostemium in the S1 stage, while *DchYABBY6* showed high expression in the ovary, sepal, and petal in the S1 stage.

### 2.8. The qRT-PCR Analysis of YABBY Genes in D. chrysotoxum

**Figure 8:**
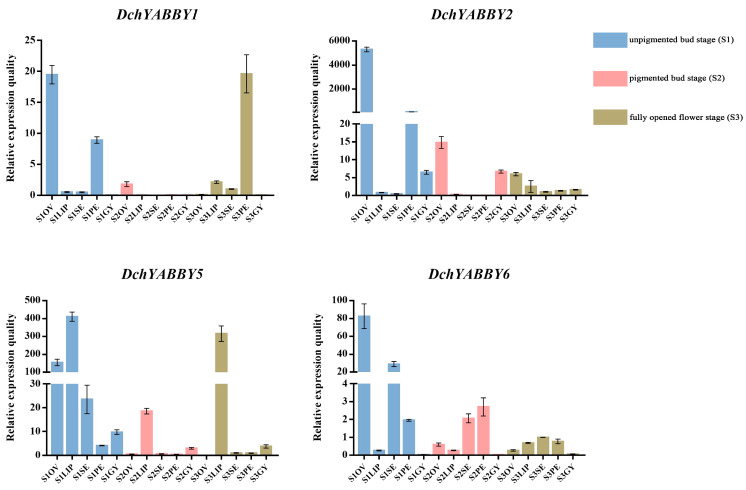
Real-time fluorescence quantitative expression analysis of *DchYABBY*s in flower development. The blue bars represent the relative expression of five flower parts in the S1 stage, the pink bars represent the relative expression of five flower parts in the S2 stage, the olive-green bars represent the relative expression of five flower parts in the S3 stage. The raw data are listed in Supplementary Table S5.

To investigate the expression patterns of the *DchYABBY* genes from the different subfamilies in five flower parts during flower development, *DchYABBY1* (INO), *DchYABBY2* (CRC/DL), *DchYABBY5* (FIL/YAB3), and *DchYABBY6* (YAB2) were selected for qRT-PCR analysis (Figure 8). The results show that the four genes were involved in ovary growth during the S1 stage, with *DchYABBY2* showing a significantly higher expression compared to the other three genes. Moreover, *DchYABBY2* exhibited sustained expression in the ovary during the subsequent two stages, highlighting its vital role in governing ovary development. The highest expression of *DchYABBY5* was found to be in the lip during the S1 stage, followed by a decrease in the S2 stage, and then an increase in the S3 stage. *DchYABBY2*, *5* exhibited continuous expression throughout the development of the gynostemium. During the S1 stage, *DchYABBY5*,*6* exhibited predominant expression in the sepal, and *DchYABBY2* displayed high expression in the petal. *DchYABBY1* showed significant expression in the petal in the S3 stage.

## 3. Discussion

YABBY transcription factors widely exist in spermatophytes, which contain two conserved domains: the C2C2 zinc-finger domain at the N-terminus and the YABBY domain at the C-terminus [6,7]. In this study, six *DchYABBY*s, nine *DhuYABBY*s, and nine *DnoYABBY*s were identified from the three *Dendrobium* species genomes. On the basis of previous research on YABBY genes in orchids, the number of YABBY genes in *Dendrobium* orchids is similar to other orchid species, such as *Cymbidium ensifolium* (7), *Cymbidium goeringii* (9), *Cymbidium sinense* (8), *Dendrobium catenatum* (8), *Gastrodia elata* (5), *Apostasiashenzenica* (6), *Phalaenopsis equestris* (8), *Platanthera zijinensis* (7), *Platanthera guangdongensis* (6), *Vanilla shenzhenica* (7), and *Vanilla pompona* (7) [27,28]. However, the number of YABBY genes in *Zea mays* (13) [29], *Gossypium arboreum* (12), *Gossypium raimondii* (12), *Gossypium hirsutum* (23) [14], and *Triticum aestivum* (20) [30] is more than that in the *Dendrobium* species, which demonstrates that genome size, chromosome number, and gene duplication are responsible for the differences.

In this study, the phylogenetic tree was constructed using six orchid species (four *Dendrobium* species, *A. shenzenica*, and *P. equestris*) and two model plants (*A. thaliana* and *O. sativa*). The results show a total of 43 orchid YABBY genes are classified into four subfamilies (CRC/DL, INO, YAB2, and FIL/YAB3), and no orchid YABBY gene was found in the YAB5 subfamily. For further confirmation, we combined the classification of other orchid YABBY genes, including *V. shenzhenica*, *V. pompona*, *C. ensifolium*, *C.goeringii*, and *C.sinense* [27,28], and found that none of these YABBY genes belonged to the YAB5 clade. Moreover, the results are consistent with some other monocot species. In *Phyllostachys edulis*, *PeYABBY*s were classified into four subfamilies (except YAB5) [31], while the *AcYABBY*s from pineapple were divided to three subfamilies—FIL/YAB3, CRC, and YAB2 [32]. Based on a phylogenetic analysis, all 20 *TaYABBY*s from wheat were classified into four clades: FIL, YAB2, INO, and CRC [33]. Due to the low homology of YAB2 and YAB5 in the Zingiberales, De Almeida believed that YAB2 and YAB5 were separated after monocots and eudicots were differentiated [34]. Further, a previous discovery held that the YAB5 gene was lost in monocot plants, and only occurred in basal angiosperms and eudicot. On the basis of these results, we believe that monocot plants might lack the YAB5 clade. It is noteworthy that each of the three *Dendrobium* species contained only one member in the INO subfamily, which is consistent with other orchids [27]. Thus, the INO subfamily is considered to be highly conserved in Orchidaceae.

The gene structure results show that each subfamily contains a unique conserved motif. Motifs 9, 6, 10, and 7 were specific to FIL/YAB3, YAB2, INO, and CRC/DL, respectively, implying that each subfamily might have certain special functions which set them apart from other subfamilies. The number of introns and exons in the same clade were similar. Almost all the YABBY members in the YAB2 clade had six exons and five introns, while all the other three subgroups had seven exons and six introns. In addition, FIL/YAB3-like, INO-like, and CRC/DL-like genes exhibited a higher number of exons and introns compared to YAB2-like genes, which revealed that YABBY genes from the former three clades were more conserved than those in the YAB2 clade. As shown in Figure 3, it is obvious that the YABBY domain is more conserved than the C2C2 domain in the three *Dendrobium* species. Overall, these findings strongly suggest that YABBY genes are relatively conserved during evolution.

Gene duplication has contributed significantly to the novelty and diversification of plants and has emerged as strong force for gene family expansion [35,36,37]. *D. chrysotoxum* has experienced two whole-genome duplication (WGD) events [24] and at least two WGD events have occurred in *D. huoshanense* and *D. nobile* since ancient times [26,38]. These events have resulted in a big difference in the distribution of YABBY genes on chromosomes and have led to differences in the number of YABBY genes among the three *Dendrobium* species. There were one, two, and two pairs of segmentally duplicated YABBY genes in *D. chrysotoxum*, *D. huoshanense*, and *D. nobile,* respectively, and one pair of tandem duplicated genes on Chr06 in *D. chrysotoxum*, suggesting that gene duplication is instrumental in the YABBY gene family in *Dendrobium*. In order to gain a deeper understanding of the evolutionary dynamics within the *Dendrobium* species, we calculated *Ka* (nonsynonymous substitution), *Ks* (synonymous substitution), and *Ka/Ks* (evolutionary selection pressure) values. The result of the *Ka/Ks* value revealed that five pairs of YABBY genes were lower than one and had experienced purifying selection.

The prediction of cis-acting elements at the transcriptional level is conductive to regulating gene expression [39]. In this study, we found that in the promoter regions of *Dendrobium* YABBY genes, MeJA-responsive elements (46/241) occurred most frequently, followed by ARE elements (39/241). As an important cellular regulator, Methyl jasmonate (MeJA) can alleviate environmental stresses, such as salt stress, drought, and low temperature, during plant developmental processes [40,41]. Anaerobiosis in plants plays a vital role in overcoming the oxygen deficits caused by flooding [42]. Thus, we suggest that YABBY genes in *Dendrobium* may be involved in defense responses against variable environments.

Combining the results of the FPKM values and qRT-PCR analysis, we found that *DchYABBY*s were strongly expressed in the unpigmented bud stage, which was basically consistent with the expression levels of YABBY genes of *D. officinale* [43]. Furthermore, *DchYABBY2* from the CRC subfamily exhibited remarkably high expression levels in the ovary across all three stages. Its expression surpassed all the rest of the genes expressing during the same developmental stage, indicating the crucial role of *DchYABBY2* in the formation and development of the ovary. This finding aligns with the established understanding that CRC serves as a vital regulatory factor in carpel development in *Arabidopsis* [44]. Previous studies have provided evidence that FIL plays a crucial role in regulating anthocyanin synthesis in *Arabidopsis* [45]. Moreover, the lip of *D. chrysotoxum* exhibited the accumulation of purple pigments [46], which correlates with the elevated expression of *DchYABBY5* in the lip, and is a member of FIL subfamily. Interestingly, the expression pattern of *DchYABBY5* showed a distinctive “high-low-high” pattern. We believe that *DchYABBY5* might participate in lip development during the non-pigmented bud stage and undergo a functional transition to contribute to the formation of purple spots during the pigmented bud stage and fully opened flower stage. According to the floral mRNA expression patterns of YAB2 in *Arabidopsis*, the expression of the YAB2 family was found in sepal, petal, and carpel primordia, which is consistent with our results for *DchYABBY6*. During the unpigmented bud stage, *DchYABBY6* participated in sepal formation and *DchYABBY2* had a significant regulatory role in petal development. However, *DchYABBY1* from the INO subfamily emerged as the dominant factor in regulating petal development during the fully opened flower period. Therefore, we believe that the petal is successively regulated by *DchYABBY2*, *1* during flower development. *DchYABBY2* and *DchYABBY5* exhibited sustained expression during gynostemium development, indicating their potential involvement in this process.

## 4. Materials and Methods

### 4.1. Data Sources

The genome sequences and annotation files of *D. chrysotoxum* and *D. nobile* were downloaded from the National Center for Biotechnology Information (NCBI, https://www.ncbi.nlm.nih.gov/, accessed on 20 November 2022) (PRJNA664445, PRJNA725550) and the *D. huoshanense* files were downloaded from the China Nucleotide Sequence Archive (CNSA, https://ftp.cngb.org/, accessed on 20 November 2022) (CNA0014590). The YABBY protein sequence files of *A. thaliana* were obtained from the Arabidopsis Information Resource (TAIR, https://www.arabidopsis.org/, accessed on 20 November 2022), the *O. sativa* subsp. *Indica* files were obtained from the Plant Transcription Factor Database (PlantTFDB, http://planttfdb.gao-lab.org/, accessed on 20 November 2022), and the *Apostasia shenzhenica*, *D. catenatum*, and *Phalaenopsis equestris* data were downloaded from NCBI (PRJNA310678, PRJNA262478, and PRJNA382149).

### 4.2. Identification and Physicochemical Properties of the YABBY Gene Family

To identify potential YABBY genes in *Dendrobium*, six *Arabidopsis* YABBY genes were used as probes in a BLAST search in TBtools v1.120 software [47]. The Hidden Markov Model (HMM) profile of the YABBY conserved domain (PF04690) from InterPro (https://www.ebi.ac.uk/interpro/, accessed on 23 November 2022) was used to further identify YABBY genes in *Dendrobium* using the Simple HMM Search in Tbtools. All the potential YABBY genes were confirmed with the NCBI CD-Search (https://www.ncbi.nlm.nih.gov/Structure/cdd/wrpsb.cgi, accessed on 24 November 2022) and SMART program (https://smart.embl.de/, accessed on 24 November 2022). Incomplete and redundant protein sequences were removed manually. Protein analyses of amino acid (aa), isoelectric point (pI), molecular weight (MW), hydrophilic large average (GRAVY), instability index (II), and fat index (AI) were performed through the online software ExPASy 3.0 (https://www.expasy.org/, accessed on 28 November 2022) [48].

### 4.3. Phylogenetic Analysis of YABBY Genes

The amino acid sequences of six YABBY proteins of *D. chrysotoxum*, nine YABBY proteins of *D. huoshanense*, nine YABBY proteins of *D. nobile*, six YABBY proteins of *A. thaliana*, seven YABBY proteins of *O. sativa*, four YABBY proteins of *A. shenzhenica*, seven YABBY proteins of *D. catenatum*, and eight YABBY proteins of *P*. *equestris* were introduced into the MEGA 7.0 software. The total 56 sequences were aligned using ClustalW with the default parameters. To construct a neighbor-joining (NJ) phylogenetic tree of the YABBYs, the bootstrap method was performed with 500 replicates and the partial deletion was set to 50%. For better visualization, the NJ phylogenetic tree was processed using the online software Evolview 3.0 (http://www.evolgenius.info/evolview/#/treeview, accessed on 12 December 2022).

### 4.4. Gene Structure and Conserved Motif Analysis of YABBY Genes

The conserved domains of the YABBY genes were predicted using NCBI’s conserved domain database CDD (https://www.ncbi.nlm.nih.gov/cdd, accessed on 20 November 2022). Moreover, Multiple Em for Motif Elicitation (MEME, https://meme-suite.org/meme/tools/meme, accessed on 20 November 2022) was used to analyze the conserved motifs of the YABBY genes in *Dendrobium*. Except for the maximum number of motifs, which was set at 10, the other parameters of MEME were kept at the defaults. The overall comparative maps of the NJ phylogenetic tree, conserved protein motifs, and gene structure were integrated through Tbtools.

### 4.5. Analysis of YABBY Genes Promoter Sequences

Tbtools was used to obtain a 2000 bp sequence upstream of the YABBY genes in *Dendrobium* as the start codon. To predict the *cis*-acting elements, elements in the promoter region of the YABBY genes in *Dendrobium* were analyzed using PlantCARE (https://bioinformatics.psb.ugent.be/webtools/plantcare/html/, accessed on 20 November 2022). Then Excel was used for data processing and Tbtools was used for visualization.

### 4.6. Chromosomal Localization and Synteny Analysis of YABBY Genes

According to the genome files and annotation files for *D. chrysotoxum*, *D. huoshanense*, and *D. nobile*, the chromosomal localizations of the YABBY genes in *Dendrobium* were visualized through Tbtools. Further, the genome data of the three *Dendrobium* species were compared to themselves for collinearity analysis using the One Step MCScanx program in Tbtools. The duplication patterns of *DchYABBY*s, *DhuYABBY*s, and *DnoYABBY*s were visualized using the Advance Circos in Tbtools. Meanwhile, the *Ka*, *Ks,* and *Ka/Ks* values were calculated using the Simple *Ka/Ks* Calculator in Tbtools.

### 4.7. Expression Analysis and RT‒qPCR

The plant materials used in this study were from the National Orchid Germplasm Resources of Fujian Agriculture and Forestry University, Fuzhou, China. All the flower parts of *D. chrysotoxum* were sampled, frozen using liquid nitrogen, and stored in a refrigerator at −80 °C. The total RNA was extracted from the different parts (sepal, petal, lip, ovary, and gynostemium) of *D. chrysotoxum* in three developmental stages, including unpigmented bud (S1), pigmented bud (S2), and fully opened flower (S3), using a FastPure Plant Total RNA Isolation Kit (for polysaccharide- and polyphenol-rich tissues) (Vazyme Biotech Co, Ltd., Nanjing, China). Transcriptome sequencing and library construction were completed by Bgi Genomics Co., Ltd. (Shenzhen, China). Then, clean reads were aligned to the assembled genome using Bowtie2 2.2.9 software. Calculations of the gene expression level of each sample were performed using the software RSEMv1.2.8 to obtain the fragments per kilobase of transcript per million fragments (FPKM) values. Finally, a heatmap representing the expression levels was generated using Tbtools based on the FPKM values.

Then we employed a Reverse Transcript Kit PrimerScript^®®^ RT reagent Kit with gDNA Eraser (TaKaRa, Dalian, China) for reverse transcription to remove the contaminated genomic DNA and generate cDNA. TB Green^®®^ Premix Ex Taq™ II (Tli RnaseH Plus) was used for a qRT-PCR analysis on an ABI 7500 Real-Time System. The RT-qPCR conditions were 20 s at 95 °C in the holding stage, and then 40 cycles of 3 s at 95 °C and 30 s at 60 °C in the cycling stage. The experimental setup utilized 96-well plates with a 20 μL reaction system in each well, and three biological replicates were performed in this study. Primers were designed using Primer Premier 5 software, and we selected *Maker75111* as the reference gene (Supplementary Table S5). The relative expressions of the target genes were calculated using the 2^−∆∆CT^ method and Graphpad prism 7.0 was used for normalization.

## 5. Conclusions

A total of six *DchYABBY*s, nine *DhuYABBY*s, and nine *DnoYABBY*s were identified and classified into four subfamilies. The genome-wide identification, phylogeny, functional classification, gene structure, motif composition, and chromosomal localization of the YABBY genes of the three *Dendrobium* species were carried out. Our results illustrate the dynamic transcription patterns of *DchYABBY*s in different flower parts during flower development. Notably, *DchYABBY2* played an indispensable role in both ovary development and early-stage petal development. *DchYABBY5* was essential for lip development, while *DchYABBY6* was crucial for sepal formation in the early stages. *DchYABBY1* primarily regulated the blooming sepals. *DchYABBY2* and *DchYABBY5* were potentially involved in the development of the gynostemium. These findings provide crucial information for future studies on the functional roles and regulatory mechanisms of the YABBY genes in the *Dendrobium* species in different flower parts throughout the various stages of flower development.

## Data Availability

The genome sequence and annotation files for *D. chrysotoxum* and *D. nobile* were downloaded from the National Center for Biotechnology Information (NCBI, https://www.ncbi.nlm.nih.gov/, accessed on 20 November 2022) (PRJNA664445, PRJNA725550), and those for *D. huoshanense* were downloaded from the China Nucleotide Sequence Archive (CNSA, https://ftp.cngb.org/, accessed on 20 November 2022) (CNA0014590).

## Supplementary Materials

The supporting information can be downloaded at: https://www.mdpi.com/article/10.3390/ijms241210165/s1.
